# Crystal structure of cyclic tris­(ferrocene-1,1′-di­yl)

**DOI:** 10.1107/S1600536814017346

**Published:** 2014-08-01

**Authors:** Ruslan Shekurov, Vasili Miluykov, Olga Kataeva, Artem Tufatullin, Oleg Sinyashin

**Affiliations:** a A. E. Arbuzov Institute of Organic and Physical Chemistry, Arbuzov Str. 8, 420088 Kazan, Russian Federation

**Keywords:** crystal structure, ferrocene, eclipsed conformation, C—H⋯π inter­actions

## Abstract

The mol­ecular structure of the trinuclear title compound, [Fe_3_(C_10_H_8_)_3_] {systematic name: tris­[μ-(η^5^:η^5^)-1,1′-bi­cyclo­penta­dien­yl]tri­iron(II)}, consists of three ferrocene subunits (each with an eclipsed conformation) that are condensed *via* C—C bonds of the fulvalene moieties into a cyclic trimer. The angles between the planes of the cyclo­penta­dienyl (Cp) rings within the three fulvalene moieties are 76.1 (3), 80.9 (3) and 81.7 (3)°. In the crystal, C—H⋯π inter­actions between neighbouring mol­ecules lead to the cohesion of the structure.

## Related literature   

The title compound was obtained as a side product during the synthesis of (ferrocene-1,1′-di­yl)bis­(*H*-phosphinic acids) (Shekurov *et al.*, 2014 ▶). In the mol­ecular structure of the related binuclear ferrocene derivative bis­(fulvalene)­diiron (Churchill & Wormald, 1969 ▶), the Cp rings of the fulvalene moieties are coplanar.
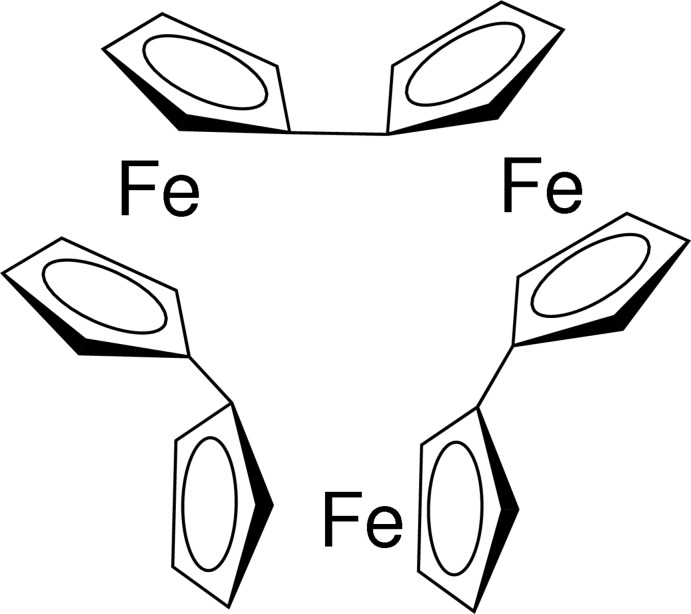


## Experimental   

### Crystal data  

[Fe_3_(C_10_H_8_)_3_]
*M* *_r_* = 552.04
Triclinic, 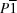

*a* = 9.9006 (16) Å
*b* = 10.5544 (17) Å
*c* = 12.448 (3) Å
α = 109.197 (5)°
β = 100.205 (5)°
γ = 108.940 (3)°
*V* = 1101.7 (4) Å^3^
*Z* = 2
Mo *K*α radiation
μ = 1.97 mm^−1^
*T* = 296 K
0.30 × 0.25 × 0.20 mm

### Data collection  

Bruker APEXII CCD diffractometer
Absorption correction: multi-scan (*SADABS*; Bruker, 2004 ▶) *T*_min_ = 0.590, *T*_max_ = 0.695
14158 measured reflections
4270 independent reflections
2958 reflections with *I* > 2σ(*I*)
*R*_int_ = 0.070

### Refinement  

*R*[*F* ^2^ > 2σ(*F* ^2^)] = 0.051
*wR*(*F* ^2^) = 0.076
*S* = 1.31
4270 reflections
298 parameters
H-atom parameters constrained
Δρ_max_ = 0.65 e Å^−3^
Δρ_min_ = −0.65 e Å^−3^

### 

Data collection: *APEX2* (Bruker, 2004 ▶); cell refinement: *SAINT* (Bruker, 2004 ▶); data reduction: *SAINT*; program(s) used to solve structure: *SIR2004* (Burla *et al.*, 2005 ▶); program(s) used to refine structure: *SHELXL97* (Sheldrick, 2008 ▶); molecular graphics: *Mercury* (Macrae *et al.*, 2006 ▶); software used to prepare material for publication: *publCIF* (Westrip, 2010 ▶).

## Supplementary Material

Crystal structure: contains datablock(s) I, mil27. DOI: 10.1107/S1600536814017346/wm5035sup1.cif

Structure factors: contains datablock(s) I. DOI: 10.1107/S1600536814017346/wm5035Isup2.hkl

Supporting information file. DOI: 10.1107/S1600536814017346/wm5035Isup3.cdx

**Fig. 1.**
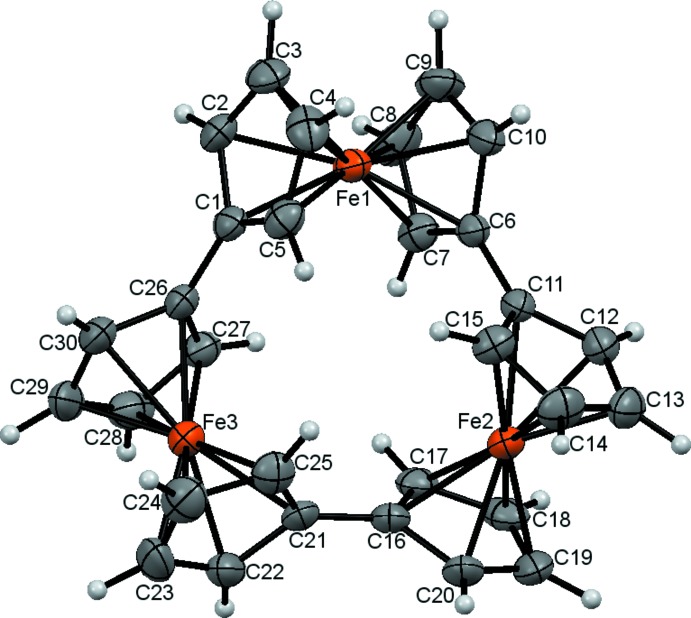
The molecular structure of the title compound, with displacement ellipsoids displayed at the 50% probability level.

. DOI: 10.1107/S1600536814017346/wm5035fig1.tif
The mol­ecular structure of the title compound, with displacement ellipsoids displayed at the 50% probability level.

CCDC reference: 1016442

Additional supporting information:  crystallographic information; 3D view; checkCIF report

## Figures and Tables

**Table 1:** Hydrogen-bond geometry (Å, °) *Cg*1 and *Cg*4 are the centroids of the C1–C5 and C16–C20 rings, respectively.

*D*—H⋯*A*	*D*—H	H⋯*A*	*D*⋯*A*	*D*—H⋯*A*
C30—H30⋯*Cg*1^i^	0.98	2.89	3.668 (5)	137
C28—H28⋯*Cg*4^ii^	0.98	2.70	3.601 (6)	153

Symmetry codes: (i) 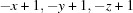; (ii) 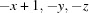.

## supplementary crystallographic information

### S1. Experimental

The title compound, [1,1'-Fc]_3_ (Fc = ferrocene), was obtained as a side product during the synthesis of (ferrocene-1,1'-diyl)bis(*H*-phosphonic acids) (Shekurov *et al.*, 2014) by reaction of 1,1'-dilithiumferrocene, Li_2_Fc, with bis(diethylamino)chlorophosphite (NEt_2_)_2_ClP in hexane. Orange crystals of the title compound were obtained on standing of solutions of 1,1'-Fc(P(NEt_2_)_2_)_2_ in hexane.

### S2. Refinement

H atoms were positioned geometrically and refined using a riding model, with C—H = 0.98 Å and with *U*_iso_(H) = 1.2*U*_eq_(C).

### Crystal data

**Table:**

[Fe_3_(C_10_H_8_)_3_]	*Z* = 2
*M**_r_* = 552.04	*F*(000) = 564
Triclinic, *P*1	*D*_x_ = 1.664 Mg m^−^^3^
Hall symbol: -P 1	Mo *K*α radiation, λ = 0.71073 Å
*a* = 9.9006 (16) Å	Cell parameters from 3519 reflections
*b* = 10.5544 (17) Å	θ = 2.2–28.4°
*c* = 12.448 (3) Å	µ = 1.97 mm^−^^1^
α = 109.197 (5)°	*T* = 296 K
β = 100.205 (5)°	Prism, orange
γ = 108.940 (3)°	0.30 × 0.25 × 0.20 mm
*V* = 1101.7 (4) Å^3^	

### Data collection

**Table:**

Bruker APEXII CCD diffractometer	4270 independent reflections
Radiation source: fine-focus sealed tube	2958 reflections with *I* > 2σ(*I*)
Graphite monochromator	*R*_int_ = 0.070
ω scans	θ_max_ = 26.0°, θ_min_ = 1.8°
Absorption correction: multi-scan (*SADABS*; Bruker, 2004)	*h* = −12→12
*T*_min_ = 0.590, *T*_max_ = 0.695	*k* = −13→13
14158 measured reflections	*l* = −15→15

### Refinement

**Table:**

Refinement on *F*^2^	Primary atom site location: structure-invariant direct methods
Least-squares matrix: full	Secondary atom site location: difference Fourier map
*R*[*F*^2^ > 2σ(*F*^2^)] = 0.051	Hydrogen site location: inferred from neighbouring sites
*wR*(*F*^2^) = 0.076	H-atom parameters constrained
*S* = 1.31	*w* = 1/[σ^2^(*F*_o_^2^) + (0.*P*)^2^] where *P* = (*F*_o_^2^ + 2*F*_c_^2^)/3
4270 reflections	(Δ/σ)_max_ < 0.001
298 parameters	Δρ_max_ = 0.65 e Å^−^^3^
0 restraints	Δρ_min_ = −0.65 e Å^−^^3^

### Special details

**Table:**

Geometry. All e.s.d.'s (except the e.s.d. in the dihedral angle between two l.s. planes) are estimated using the full covariance matrix. The cell e.s.d.'s are taken into account individually in the estimation of e.s.d.'s in distances, angles and torsion angles; correlations between e.s.d.'s in cell parameters are only used when they are defined by crystal symmetry. An approximate (isotropic) treatment of cell e.s.d.'s is used for estimating e.s.d.'s involving l.s. planes.
Refinement. Refinement of *F*^2^ against ALL reflections. The weighted *R*-factor *wR* and goodness of fit *S* are based on *F*^2^, conventional *R*-factors *R* are based on *F*, with *F* set to zero for negative *F*^2^. The threshold expression of *F*^2^ > σ(*F*^2^) is used only for calculating *R*-factors(gt) *etc*. and is not relevant to the choice of reflections for refinement. *R*-factors based on *F*^2^ are statistically about twice as large as those based on *F*, and *R*- factors based on ALL data will be even larger.

### Fractional atomic coordinates and isotropic or equivalent isotropic displacement parameters (Å^2^)

**Table:**

	*x*	*y*	*z*	*U*_iso_*/*U*_eq_	
C1	0.6533 (4)	0.5589 (4)	0.3621 (4)	0.0316 (10)	
C2	0.6144 (5)	0.6798 (4)	0.3643 (4)	0.0385 (11)	
H2	0.5183	0.6725	0.3192	0.046*	
C3	0.7420 (5)	0.8118 (4)	0.4438 (4)	0.0438 (12)	
H3	0.7486	0.9119	0.4633	0.053*	
C4	0.8562 (5)	0.7750 (4)	0.4912 (4)	0.0443 (12)	
H4	0.9559	0.8446	0.5488	0.053*	
C5	0.8019 (5)	0.6200 (4)	0.4408 (4)	0.0371 (11)	
H5	0.8589	0.5639	0.4565	0.044*	
C6	0.9417 (4)	0.6495 (4)	0.2144 (4)	0.0308 (10)	
C7	0.7931 (4)	0.5911 (4)	0.1360 (4)	0.0349 (10)	
H7	0.7313	0.4869	0.0834	0.042*	
C8	0.7472 (5)	0.7075 (5)	0.1485 (4)	0.0461 (12)	
H8	0.6496	0.6985	0.1046	0.055*	
C9	0.8672 (6)	0.8386 (5)	0.2338 (5)	0.0521 (14)	
H9	0.8680	0.9372	0.2596	0.062*	
C10	0.9859 (5)	0.8044 (4)	0.2741 (4)	0.0417 (12)	
H10	1.0835	0.8752	0.3344	0.050*	
C11	1.0423 (4)	0.5741 (4)	0.2255 (4)	0.0302 (10)	
C12	1.1292 (4)	0.5442 (4)	0.1481 (4)	0.0360 (11)	
H12	1.1255	0.5618	0.0753	0.043*	
C13	1.2229 (4)	0.4864 (4)	0.1958 (4)	0.0435 (12)	
H13	1.2957	0.4572	0.1617	0.052*	
C14	1.1941 (5)	0.4781 (4)	0.2998 (4)	0.0459 (12)	
H14	1.2430	0.4419	0.3512	0.055*	
C15	1.0833 (4)	0.5324 (4)	0.3187 (4)	0.0366 (11)	
H15	1.0408	0.5384	0.3846	0.044*	
C16	0.8243 (4)	0.1780 (4)	0.1464 (4)	0.0323 (10)	
C17	0.7861 (4)	0.2140 (4)	0.0490 (4)	0.0362 (11)	
H17	0.7038	0.2437	0.0309	0.043*	
C18	0.8882 (5)	0.2040 (4)	−0.0165 (4)	0.0429 (12)	
H18	0.8874	0.2229	−0.0885	0.051*	
C19	0.9902 (5)	0.1617 (4)	0.0398 (4)	0.0465 (12)	
H19	1.0729	0.1449	0.0134	0.056*	
C20	0.9525 (4)	0.1453 (4)	0.1404 (4)	0.0409 (12)	
H20	1.0047	0.1157	0.1958	0.049*	
C21	0.7380 (4)	0.1526 (4)	0.2280 (4)	0.0318 (10)	
C22	0.6042 (4)	0.0233 (4)	0.1953 (5)	0.0440 (12)	
H22	0.5492	−0.0544	0.1141	0.053*	
C23	0.5647 (5)	0.0263 (5)	0.2986 (5)	0.0543 (14)	
H23	0.4787	−0.0492	0.3023	0.065*	
C24	0.6711 (5)	0.1558 (5)	0.3958 (5)	0.0511 (13)	
H24	0.6718	0.1867	0.4793	0.061*	
C25	0.7783 (5)	0.2333 (4)	0.3531 (4)	0.0391 (11)	
H25	0.8646	0.3280	0.4018	0.047*	
C26	0.5445 (4)	0.4019 (4)	0.2984 (4)	0.0325 (10)	
C27	0.4946 (4)	0.3110 (4)	0.1753 (4)	0.0374 (11)	
H27	0.5360	0.3358	0.1153	0.045*	
C28	0.3759 (5)	0.1773 (4)	0.1525 (5)	0.0478 (12)	
H28	0.3196	0.0944	0.0739	0.057*	
C29	0.3508 (5)	0.1844 (5)	0.2608 (5)	0.0498 (13)	
H29	0.2741	0.1073	0.2714	0.060*	
C30	0.4563 (5)	0.3216 (4)	0.3530 (4)	0.0414 (11)	
H30	0.4651	0.3567	0.4383	0.050*	
Fe1	0.79971 (6)	0.69662 (6)	0.30904 (5)	0.03287 (17)	
Fe2	1.00395 (6)	0.35453 (6)	0.15645 (5)	0.03206 (17)	
Fe3	0.56465 (6)	0.20506 (6)	0.27057 (6)	0.03564 (18)	

### Atomic displacement parameters (Å^2^)

**Table:**

	*U*^11^	*U*^22^	*U*^33^	*U*^12^	*U*^13^	*U*^23^
C1	0.038 (3)	0.031 (2)	0.032 (3)	0.018 (2)	0.019 (2)	0.013 (2)
C2	0.038 (3)	0.040 (2)	0.047 (3)	0.024 (2)	0.023 (2)	0.017 (2)
C3	0.056 (3)	0.032 (2)	0.043 (3)	0.021 (2)	0.023 (3)	0.009 (2)
C4	0.042 (3)	0.044 (3)	0.030 (3)	0.008 (2)	0.008 (2)	0.007 (2)
C5	0.042 (3)	0.043 (3)	0.032 (3)	0.023 (2)	0.014 (2)	0.016 (2)
C6	0.039 (3)	0.028 (2)	0.034 (3)	0.016 (2)	0.020 (2)	0.016 (2)
C7	0.043 (3)	0.038 (2)	0.031 (3)	0.021 (2)	0.014 (2)	0.016 (2)
C8	0.055 (3)	0.066 (3)	0.044 (3)	0.043 (3)	0.020 (3)	0.034 (3)
C9	0.077 (4)	0.040 (3)	0.067 (4)	0.037 (3)	0.039 (3)	0.034 (3)
C10	0.051 (3)	0.029 (2)	0.047 (3)	0.014 (2)	0.027 (3)	0.014 (2)
C11	0.032 (2)	0.025 (2)	0.034 (3)	0.0104 (19)	0.014 (2)	0.011 (2)
C12	0.036 (3)	0.033 (2)	0.041 (3)	0.012 (2)	0.016 (2)	0.015 (2)
C13	0.028 (3)	0.040 (3)	0.058 (4)	0.015 (2)	0.014 (3)	0.015 (3)
C14	0.042 (3)	0.051 (3)	0.048 (3)	0.026 (2)	0.007 (3)	0.021 (3)
C15	0.040 (3)	0.038 (2)	0.034 (3)	0.020 (2)	0.011 (2)	0.013 (2)
C16	0.037 (3)	0.024 (2)	0.040 (3)	0.016 (2)	0.014 (2)	0.014 (2)
C17	0.039 (3)	0.022 (2)	0.041 (3)	0.012 (2)	0.009 (2)	0.007 (2)
C18	0.065 (3)	0.025 (2)	0.035 (3)	0.016 (2)	0.019 (3)	0.008 (2)
C19	0.056 (3)	0.031 (2)	0.060 (4)	0.022 (2)	0.035 (3)	0.015 (2)
C20	0.044 (3)	0.031 (2)	0.061 (4)	0.023 (2)	0.023 (3)	0.025 (2)
C21	0.033 (2)	0.029 (2)	0.041 (3)	0.020 (2)	0.015 (2)	0.016 (2)
C22	0.038 (3)	0.029 (2)	0.067 (4)	0.016 (2)	0.020 (3)	0.018 (2)
C23	0.052 (3)	0.052 (3)	0.088 (5)	0.026 (3)	0.041 (3)	0.048 (3)
C24	0.064 (3)	0.061 (3)	0.052 (4)	0.035 (3)	0.028 (3)	0.036 (3)
C25	0.036 (3)	0.045 (3)	0.039 (3)	0.020 (2)	0.007 (2)	0.020 (2)
C26	0.032 (2)	0.034 (2)	0.039 (3)	0.019 (2)	0.018 (2)	0.016 (2)
C27	0.037 (3)	0.034 (2)	0.040 (3)	0.018 (2)	0.013 (2)	0.012 (2)
C28	0.037 (3)	0.035 (3)	0.061 (4)	0.016 (2)	0.005 (3)	0.011 (3)
C29	0.028 (3)	0.038 (3)	0.087 (4)	0.015 (2)	0.024 (3)	0.026 (3)
C30	0.046 (3)	0.041 (3)	0.053 (3)	0.027 (2)	0.029 (3)	0.022 (3)
Fe1	0.0387 (4)	0.0287 (3)	0.0351 (4)	0.0173 (3)	0.0150 (3)	0.0128 (3)
Fe2	0.0331 (4)	0.0287 (3)	0.0385 (4)	0.0164 (3)	0.0138 (3)	0.0139 (3)
Fe3	0.0332 (4)	0.0325 (3)	0.0452 (5)	0.0159 (3)	0.0166 (3)	0.0163 (3)

### Geometric parameters (Å, º)

**Table:**

C1—C5	1.414 (5)	C16—C17	1.411 (5)
C1—C2	1.442 (5)	C16—C20	1.427 (5)
C1—C26	1.487 (5)	C16—C21	1.474 (5)
C1—Fe1	2.062 (4)	C16—Fe2	2.066 (4)
C2—C3	1.422 (5)	C17—C18	1.415 (5)
C2—Fe1	2.046 (4)	C17—Fe2	2.058 (4)
C2—H2	0.9800	C17—H17	0.9800
C3—C4	1.407 (5)	C18—C19	1.400 (5)
C3—Fe1	2.017 (4)	C18—Fe2	2.042 (4)
C3—H3	0.9800	C18—H18	0.9800
C4—C5	1.412 (5)	C19—C20	1.415 (5)
C4—Fe1	2.039 (4)	C19—Fe2	2.019 (4)
C4—H4	0.9800	C19—H19	0.9800
C5—Fe1	2.054 (4)	C20—Fe2	2.030 (4)
C5—H5	0.9800	C20—H20	0.9800
C6—C7	1.415 (5)	C21—C25	1.413 (5)
C6—C10	1.427 (5)	C21—C22	1.433 (5)
C6—C11	1.477 (5)	C21—Fe3	2.068 (4)
C6—Fe1	2.065 (4)	C22—C23	1.402 (6)
C7—C8	1.415 (5)	C22—Fe3	2.036 (4)
C7—Fe1	2.052 (4)	C22—H22	0.9800
C7—H7	0.9800	C23—C24	1.403 (6)
C8—C9	1.404 (6)	C23—Fe3	2.029 (4)
C8—Fe1	2.024 (4)	C23—H23	0.9800
C8—H8	0.9800	C24—C25	1.414 (5)
C9—C10	1.396 (5)	C24—Fe3	2.029 (4)
C9—Fe1	2.016 (4)	C24—H24	0.9800
C9—H9	0.9800	C25—Fe3	2.059 (4)
C10—Fe1	2.032 (4)	C25—H25	0.9800
C10—H10	0.9800	C26—C27	1.404 (5)
C11—C15	1.411 (5)	C26—C30	1.432 (5)
C11—C12	1.427 (5)	C26—Fe3	2.072 (4)
C11—Fe2	2.063 (4)	C27—C28	1.413 (5)
C12—C13	1.411 (5)	C27—Fe3	2.055 (4)
C12—Fe2	2.031 (4)	C27—H27	0.9800
C12—H12	0.9800	C28—C29	1.396 (6)
C13—C14	1.398 (6)	C28—Fe3	2.032 (4)
C13—Fe2	2.023 (4)	C28—H28	0.9800
C13—H13	0.9800	C29—C30	1.416 (6)
C14—C15	1.413 (5)	C29—Fe3	2.033 (4)
C14—Fe2	2.028 (4)	C29—H29	0.9800
C14—H14	0.9800	C30—Fe3	2.033 (4)
C15—Fe2	2.051 (4)	C30—H30	0.9800
C15—H15	0.9800		
			
C5—C1—C2	107.2 (3)	C28—C27—Fe3	68.9 (2)
C5—C1—C26	128.8 (4)	C26—C27—H27	125.7
C2—C1—C26	123.5 (4)	C28—C27—H27	125.7
C5—C1—Fe1	69.6 (2)	Fe3—C27—H27	125.7
C2—C1—Fe1	68.9 (2)	C29—C28—C27	108.3 (4)
C26—C1—Fe1	132.3 (3)	C29—C28—Fe3	70.0 (3)
C3—C2—C1	107.1 (4)	C27—C28—Fe3	70.7 (2)
C3—C2—Fe1	68.4 (2)	C29—C28—H28	125.8
C1—C2—Fe1	70.1 (2)	C27—C28—H28	125.8
C3—C2—H2	126.5	Fe3—C28—H28	125.8
C1—C2—H2	126.5	C28—C29—C30	108.2 (4)
Fe1—C2—H2	126.5	C28—C29—Fe3	69.8 (2)
C4—C3—C2	108.8 (4)	C30—C29—Fe3	69.6 (2)
C4—C3—Fe1	70.5 (2)	C28—C29—H29	125.9
C2—C3—Fe1	70.6 (2)	C30—C29—H29	125.9
C4—C3—H3	125.6	Fe3—C29—H29	125.9
C2—C3—H3	125.6	C29—C30—C26	107.6 (4)
Fe1—C3—H3	125.6	C29—C30—Fe3	69.6 (2)
C3—C4—C5	107.9 (4)	C26—C30—Fe3	71.1 (2)
C3—C4—Fe1	68.9 (3)	C29—C30—H30	126.2
C5—C4—Fe1	70.4 (2)	C26—C30—H30	126.2
C3—C4—H4	126.1	Fe3—C30—H30	126.2
C5—C4—H4	126.1	C9—Fe1—C3	102.26 (17)
Fe1—C4—H4	126.1	C9—Fe1—C8	40.68 (17)
C4—C5—C1	109.0 (4)	C3—Fe1—C8	119.35 (18)
C4—C5—Fe1	69.2 (2)	C9—Fe1—C10	40.33 (15)
C1—C5—Fe1	70.2 (2)	C3—Fe1—C10	118.61 (16)
C4—C5—H5	125.5	C8—Fe1—C10	67.99 (18)
C1—C5—H5	125.5	C9—Fe1—C4	119.14 (19)
Fe1—C5—H5	125.5	C3—Fe1—C4	40.58 (15)
C7—C6—C10	106.2 (4)	C8—Fe1—C4	155.12 (17)
C7—C6—C11	128.7 (3)	C10—Fe1—C4	106.03 (17)
C10—C6—C11	124.7 (4)	C9—Fe1—C2	118.76 (17)
C7—C6—Fe1	69.4 (2)	C3—Fe1—C2	40.98 (15)
C10—C6—Fe1	68.4 (2)	C8—Fe1—C2	105.85 (17)
C11—C6—Fe1	132.1 (3)	C10—Fe1—C2	154.35 (15)
C6—C7—C8	108.7 (4)	C4—Fe1—C2	68.55 (17)
C6—C7—Fe1	70.4 (2)	C9—Fe1—C7	68.13 (17)
C8—C7—Fe1	68.7 (2)	C3—Fe1—C7	157.90 (17)
C6—C7—H7	125.6	C8—Fe1—C7	40.63 (14)
C8—C7—H7	125.6	C10—Fe1—C7	67.65 (16)
Fe1—C7—H7	125.6	C4—Fe1—C7	161.48 (16)
C9—C8—C7	107.8 (4)	C2—Fe1—C7	124.82 (17)
C9—C8—Fe1	69.4 (3)	C9—Fe1—C5	157.23 (19)
C7—C8—Fe1	70.7 (2)	C3—Fe1—C5	68.08 (16)
C9—C8—H8	126.1	C8—Fe1—C5	162.02 (18)
C7—C8—H8	126.1	C10—Fe1—C5	124.78 (17)
Fe1—C8—H8	126.1	C4—Fe1—C5	40.37 (14)
C10—C9—C8	108.2 (4)	C2—Fe1—C5	68.22 (16)
C10—C9—Fe1	70.5 (2)	C7—Fe1—C5	127.87 (15)
C8—C9—Fe1	70.0 (2)	C9—Fe1—C1	157.37 (18)
C10—C9—H9	125.9	C3—Fe1—C1	68.77 (15)
C8—C9—H9	125.9	C8—Fe1—C1	124.38 (18)
Fe1—C9—H9	125.9	C10—Fe1—C1	162.19 (16)
C9—C10—C6	109.0 (4)	C4—Fe1—C1	68.23 (16)
C9—C10—Fe1	69.2 (2)	C2—Fe1—C1	41.09 (13)
C6—C10—Fe1	70.9 (2)	C7—Fe1—C1	112.16 (15)
C9—C10—H10	125.5	C5—Fe1—C1	40.18 (14)
C6—C10—H10	125.5	C9—Fe1—C6	68.54 (16)
Fe1—C10—H10	125.5	C3—Fe1—C6	156.58 (17)
C15—C11—C12	107.0 (3)	C8—Fe1—C6	68.43 (16)
C15—C11—C6	127.6 (4)	C10—Fe1—C6	40.75 (13)
C12—C11—C6	125.1 (4)	C4—Fe1—C6	123.77 (17)
C15—C11—Fe2	69.5 (2)	C2—Fe1—C6	162.32 (16)
C12—C11—Fe2	68.4 (2)	C7—Fe1—C6	40.21 (14)
C6—C11—Fe2	132.0 (3)	C5—Fe1—C6	111.81 (15)
C13—C12—C11	108.0 (4)	C1—Fe1—C6	127.38 (14)
C13—C12—Fe2	69.3 (2)	C19—Fe2—C13	101.05 (17)
C11—C12—Fe2	70.8 (2)	C19—Fe2—C14	116.93 (18)
C13—C12—H12	126.0	C13—Fe2—C14	40.38 (16)
C11—C12—H12	126.0	C19—Fe2—C20	40.91 (15)
Fe2—C12—H12	126.0	C13—Fe2—C20	117.94 (16)
C14—C13—C12	108.3 (4)	C14—Fe2—C20	104.36 (17)
C14—C13—Fe2	70.0 (2)	C19—Fe2—C12	118.90 (17)
C12—C13—Fe2	69.9 (2)	C13—Fe2—C12	40.75 (15)
C14—C13—H13	125.8	C14—Fe2—C12	68.28 (17)
C12—C13—H13	125.8	C20—Fe2—C12	154.47 (16)
Fe2—C13—H13	125.8	C19—Fe2—C18	40.33 (15)
C13—C14—C15	108.1 (4)	C13—Fe2—C18	118.12 (18)
C13—C14—Fe2	69.6 (3)	C14—Fe2—C18	152.70 (18)
C15—C14—Fe2	70.6 (2)	C20—Fe2—C18	68.23 (17)
C13—C14—H14	125.9	C12—Fe2—C18	106.54 (17)
C15—C14—H14	125.9	C19—Fe2—C15	155.19 (18)
Fe2—C14—H14	125.9	C13—Fe2—C15	67.94 (17)
C11—C15—C14	108.5 (4)	C14—Fe2—C15	40.54 (14)
C11—C15—Fe2	70.4 (2)	C20—Fe2—C15	123.15 (17)
C14—C15—Fe2	68.9 (2)	C12—Fe2—C15	67.98 (16)
C11—C15—H15	125.7	C18—Fe2—C15	164.46 (16)
C14—C15—H15	125.7	C19—Fe2—C17	67.76 (17)
Fe2—C15—H15	125.7	C13—Fe2—C17	156.68 (18)
C17—C16—C20	106.7 (3)	C14—Fe2—C17	162.89 (17)
C17—C16—C21	128.2 (4)	C20—Fe2—C17	67.73 (16)
C20—C16—C21	124.3 (4)	C12—Fe2—C17	125.41 (17)
C17—C16—Fe2	69.7 (2)	C18—Fe2—C17	40.37 (14)
C20—C16—Fe2	68.3 (2)	C15—Fe2—C17	130.10 (16)
C21—C16—Fe2	134.5 (3)	C19—Fe2—C11	158.03 (17)
C16—C17—C18	109.0 (4)	C13—Fe2—C11	68.38 (15)
C16—C17—Fe2	70.3 (2)	C14—Fe2—C11	68.14 (16)
C18—C17—Fe2	69.2 (2)	C20—Fe2—C11	161.05 (17)
C16—C17—H17	125.5	C12—Fe2—C11	40.79 (13)
C18—C17—H17	125.5	C18—Fe2—C11	126.42 (17)
Fe2—C17—H17	125.5	C15—Fe2—C11	40.11 (14)
C19—C18—C17	107.7 (4)	C17—Fe2—C11	114.23 (15)
C19—C18—Fe2	69.0 (3)	C19—Fe2—C16	68.55 (16)
C17—C18—Fe2	70.4 (2)	C13—Fe2—C16	156.63 (17)
C19—C18—H18	126.1	C14—Fe2—C16	124.27 (17)
C17—C18—H18	126.1	C20—Fe2—C16	40.78 (13)
Fe2—C18—H18	126.1	C12—Fe2—C16	162.57 (16)
C18—C19—C20	108.4 (4)	C18—Fe2—C16	68.13 (16)
C18—C19—Fe2	70.7 (2)	C15—Fe2—C16	112.50 (16)
C20—C19—Fe2	70.0 (2)	C17—Fe2—C16	40.02 (14)
C18—C19—H19	125.8	C11—Fe2—C16	128.15 (15)
C20—C19—H19	125.8	C23—Fe3—C24	40.45 (17)
Fe2—C19—H19	125.8	C23—Fe3—C28	116.96 (19)
C19—C20—C16	108.1 (4)	C24—Fe3—C28	151.84 (18)
C19—C20—Fe2	69.1 (2)	C23—Fe3—C30	119.96 (17)
C16—C20—Fe2	71.0 (2)	C24—Fe3—C30	105.80 (18)
C19—C20—H20	126.0	C28—Fe3—C30	68.19 (18)
C16—C20—H20	126.0	C23—Fe3—C29	101.86 (17)
Fe2—C20—H20	126.0	C24—Fe3—C29	117.27 (19)
C25—C21—C22	106.5 (4)	C28—Fe3—C29	40.18 (17)
C25—C21—C16	128.6 (4)	C30—Fe3—C29	40.77 (16)
C22—C21—C16	124.2 (4)	C23—Fe3—C22	40.35 (17)
C25—C21—Fe3	69.6 (2)	C24—Fe3—C22	67.78 (19)
C22—C21—Fe3	68.4 (2)	C28—Fe3—C22	106.07 (18)
C16—C21—Fe3	133.9 (3)	C30—Fe3—C22	156.22 (16)
C23—C22—C21	108.8 (4)	C29—Fe3—C22	120.04 (16)
C23—C22—Fe3	69.5 (2)	C23—Fe3—C27	154.7 (2)
C21—C22—Fe3	70.8 (2)	C24—Fe3—C27	164.80 (18)
C23—C22—H22	125.6	C28—Fe3—C27	40.45 (14)
C21—C22—H22	125.6	C30—Fe3—C27	67.91 (16)
Fe3—C22—H22	125.6	C29—Fe3—C27	67.70 (17)
C22—C23—C24	107.9 (4)	C22—Fe3—C27	123.60 (18)
C22—C23—Fe3	70.1 (2)	C23—Fe3—C25	67.98 (17)
C24—C23—Fe3	69.8 (2)	C24—Fe3—C25	40.46 (15)
C22—C23—H23	126.1	C28—Fe3—C25	164.75 (18)
C24—C23—H23	126.1	C30—Fe3—C25	123.24 (18)
Fe3—C23—H23	126.1	C29—Fe3—C25	155.06 (19)
C23—C24—C25	108.5 (4)	C22—Fe3—C25	67.67 (17)
C23—C24—Fe3	69.8 (3)	C27—Fe3—C25	130.43 (16)
C25—C24—Fe3	70.9 (2)	C23—Fe3—C21	68.45 (16)
C23—C24—H24	125.8	C24—Fe3—C21	68.07 (16)
C25—C24—H24	125.8	C28—Fe3—C21	126.28 (19)
Fe3—C24—H24	125.8	C30—Fe3—C21	160.14 (16)
C21—C25—C24	108.4 (4)	C29—Fe3—C21	159.06 (18)
C21—C25—Fe3	70.3 (2)	C22—Fe3—C21	40.85 (14)
C24—C25—Fe3	68.6 (2)	C27—Fe3—C21	112.88 (16)
C21—C25—H25	125.8	C25—Fe3—C21	40.05 (15)
C24—C25—H25	125.8	C23—Fe3—C26	159.29 (18)
Fe3—C25—H25	125.8	C24—Fe3—C26	126.64 (18)
C27—C26—C30	107.3 (4)	C28—Fe3—C26	67.72 (16)
C27—C26—C1	128.1 (4)	C30—Fe3—C26	40.82 (14)
C30—C26—C1	124.2 (4)	C29—Fe3—C26	68.09 (15)
C27—C26—Fe3	69.5 (2)	C22—Fe3—C26	160.35 (17)
C30—C26—Fe3	68.1 (2)	C27—Fe3—C26	39.77 (14)
C1—C26—Fe3	133.4 (3)	C25—Fe3—C26	113.23 (16)
C26—C27—C28	108.6 (4)	C21—Fe3—C26	126.79 (14)
C26—C27—Fe3	70.8 (2)		
			
C5—C1—C2—C3	0.6 (4)	C14—C13—Fe2—C20	−79.0 (3)
C26—C1—C2—C3	173.9 (4)	C12—C13—Fe2—C20	161.7 (2)
Fe1—C1—C2—C3	−58.6 (3)	C14—C13—Fe2—C12	119.3 (4)
C5—C1—C2—Fe1	59.2 (3)	C14—C13—Fe2—C18	−157.9 (2)
C26—C1—C2—Fe1	−127.5 (4)	C12—C13—Fe2—C18	82.8 (3)
C1—C2—C3—C4	−0.8 (5)	C14—C13—Fe2—C15	37.9 (2)
Fe1—C2—C3—C4	−60.5 (3)	C12—C13—Fe2—C15	−81.4 (3)
C1—C2—C3—Fe1	59.6 (3)	C14—C13—Fe2—C17	−177.5 (3)
C2—C3—C4—C5	0.7 (5)	C12—C13—Fe2—C17	63.2 (5)
Fe1—C3—C4—C5	−59.8 (3)	C14—C13—Fe2—C11	81.3 (3)
C2—C3—C4—Fe1	60.5 (3)	C12—C13—Fe2—C11	−38.0 (2)
C3—C4—C5—C1	−0.3 (5)	C14—C13—Fe2—C16	−58.0 (5)
Fe1—C4—C5—C1	−59.1 (3)	C12—C13—Fe2—C16	−177.3 (4)
C3—C4—C5—Fe1	58.9 (3)	C13—C14—Fe2—C19	74.6 (3)
C2—C1—C5—C4	−0.2 (4)	C15—C14—Fe2—C19	−166.5 (2)
C26—C1—C5—C4	−173.0 (4)	C15—C14—Fe2—C13	118.9 (4)
Fe1—C1—C5—C4	58.5 (3)	C13—C14—Fe2—C20	116.4 (3)
C2—C1—C5—Fe1	−58.8 (3)	C15—C14—Fe2—C20	−124.7 (2)
C26—C1—C5—Fe1	128.5 (4)	C13—C14—Fe2—C12	−37.8 (2)
C10—C6—C7—C8	−0.6 (4)	C15—C14—Fe2—C12	81.1 (2)
C11—C6—C7—C8	−173.8 (4)	C13—C14—Fe2—C18	46.2 (5)
Fe1—C6—C7—C8	58.1 (3)	C15—C14—Fe2—C18	165.1 (3)
C10—C6—C7—Fe1	−58.7 (3)	C13—C14—Fe2—C15	−118.9 (4)
C11—C6—C7—Fe1	128.0 (4)	C13—C14—Fe2—C17	176.6 (5)
C6—C7—C8—C9	0.5 (5)	C15—C14—Fe2—C17	−64.5 (6)
Fe1—C7—C8—C9	59.7 (3)	C13—C14—Fe2—C11	−81.9 (3)
C6—C7—C8—Fe1	−59.2 (3)	C15—C14—Fe2—C11	37.0 (2)
C7—C8—C9—C10	−0.2 (5)	C13—C14—Fe2—C16	156.0 (2)
Fe1—C8—C9—C10	60.4 (3)	C15—C14—Fe2—C16	−85.2 (3)
C7—C8—C9—Fe1	−60.5 (3)	C16—C20—Fe2—C19	−118.9 (4)
C8—C9—C10—C6	−0.2 (5)	C19—C20—Fe2—C13	−73.7 (3)
Fe1—C9—C10—C6	59.9 (3)	C16—C20—Fe2—C13	167.4 (3)
C8—C9—C10—Fe1	−60.1 (3)	C19—C20—Fe2—C14	−114.7 (3)
C7—C6—C10—C9	0.5 (5)	C16—C20—Fe2—C14	126.4 (3)
C11—C6—C10—C9	174.1 (4)	C19—C20—Fe2—C12	−45.3 (5)
Fe1—C6—C10—C9	−58.9 (3)	C16—C20—Fe2—C12	−164.1 (4)
C7—C6—C10—Fe1	59.3 (3)	C19—C20—Fe2—C18	37.6 (3)
C11—C6—C10—Fe1	−127.1 (4)	C16—C20—Fe2—C18	−81.3 (3)
C7—C6—C11—C15	−106.9 (5)	C19—C20—Fe2—C15	−154.4 (3)
C10—C6—C11—C15	81.0 (5)	C16—C20—Fe2—C15	86.7 (3)
Fe1—C6—C11—C15	−10.2 (6)	C19—C20—Fe2—C17	81.3 (3)
C7—C6—C11—C12	80.3 (5)	C16—C20—Fe2—C17	−37.6 (2)
C10—C6—C11—C12	−91.8 (5)	C19—C20—Fe2—C11	−178.8 (4)
Fe1—C6—C11—C12	176.9 (3)	C16—C20—Fe2—C11	62.3 (6)
C7—C6—C11—Fe2	−11.3 (6)	C19—C20—Fe2—C16	118.9 (4)
C10—C6—C11—Fe2	176.6 (3)	C13—C12—Fe2—C19	−72.2 (3)
Fe1—C6—C11—Fe2	85.4 (5)	C11—C12—Fe2—C19	169.0 (3)
C15—C11—C12—C13	−0.6 (4)	C11—C12—Fe2—C13	−118.7 (4)
C6—C11—C12—C13	173.4 (4)	C13—C12—Fe2—C14	37.5 (3)
Fe2—C11—C12—C13	−59.6 (3)	C11—C12—Fe2—C14	−81.3 (3)
C15—C11—C12—Fe2	58.9 (3)	C13—C12—Fe2—C20	−40.1 (5)
C6—C11—C12—Fe2	−127.0 (4)	C11—C12—Fe2—C20	−158.9 (4)
C11—C12—C13—C14	0.8 (5)	C13—C12—Fe2—C18	−114.1 (3)
Fe2—C12—C13—C14	−59.7 (3)	C11—C12—Fe2—C18	127.1 (3)
C11—C12—C13—Fe2	60.5 (3)	C13—C12—Fe2—C15	81.3 (3)
C12—C13—C14—C15	−0.7 (5)	C11—C12—Fe2—C15	−37.4 (2)
Fe2—C13—C14—C15	−60.3 (3)	C13—C12—Fe2—C17	−154.3 (3)
C12—C13—C14—Fe2	59.7 (3)	C11—C12—Fe2—C17	87.0 (3)
C12—C11—C15—C14	0.2 (4)	C13—C12—Fe2—C11	118.7 (4)
C6—C11—C15—C14	−173.6 (3)	C13—C12—Fe2—C16	176.4 (5)
Fe2—C11—C15—C14	58.5 (3)	C11—C12—Fe2—C16	57.7 (6)
C12—C11—C15—Fe2	−58.3 (3)	C17—C18—Fe2—C19	118.9 (4)
C6—C11—C15—Fe2	127.9 (4)	C19—C18—Fe2—C13	72.9 (3)
C13—C14—C15—C11	0.3 (5)	C17—C18—Fe2—C13	−168.2 (2)
Fe2—C14—C15—C11	−59.4 (3)	C19—C18—Fe2—C14	40.9 (5)
C13—C14—C15—Fe2	59.7 (3)	C17—C18—Fe2—C14	159.7 (3)
C20—C16—C17—C18	0.1 (4)	C19—C18—Fe2—C20	−38.1 (2)
C21—C16—C17—C18	−170.3 (4)	C17—C18—Fe2—C20	80.8 (2)
Fe2—C16—C17—C18	58.5 (3)	C19—C18—Fe2—C12	115.4 (3)
C20—C16—C17—Fe2	−58.4 (3)	C17—C18—Fe2—C12	−125.7 (2)
C21—C16—C17—Fe2	131.3 (4)	C19—C18—Fe2—C15	−177.7 (5)
C16—C17—C18—C19	0.0 (4)	C17—C18—Fe2—C15	−58.8 (7)
Fe2—C17—C18—C19	59.1 (3)	C19—C18—Fe2—C17	−118.9 (4)
C16—C17—C18—Fe2	−59.1 (3)	C19—C18—Fe2—C11	155.7 (2)
C17—C18—C19—C20	0.0 (4)	C17—C18—Fe2—C11	−85.4 (3)
Fe2—C18—C19—C20	60.0 (3)	C19—C18—Fe2—C16	−82.2 (3)
C17—C18—C19—Fe2	−60.0 (3)	C17—C18—Fe2—C16	36.7 (2)
C18—C19—C20—C16	0.1 (5)	C11—C15—Fe2—C19	149.6 (4)
Fe2—C19—C20—C16	60.5 (3)	C14—C15—Fe2—C19	29.7 (5)
C18—C19—C20—Fe2	−60.5 (3)	C11—C15—Fe2—C13	82.2 (2)
C17—C16—C20—C19	−0.1 (4)	C14—C15—Fe2—C13	−37.7 (2)
C21—C16—C20—C19	170.7 (4)	C11—C15—Fe2—C14	119.9 (3)
Fe2—C16—C20—C19	−59.4 (3)	C11—C15—Fe2—C20	−168.0 (2)
C17—C16—C20—Fe2	59.3 (3)	C14—C15—Fe2—C20	72.1 (3)
C21—C16—C20—Fe2	−129.9 (4)	C11—C15—Fe2—C12	38.1 (2)
C17—C16—C21—C25	−117.0 (5)	C14—C15—Fe2—C12	−81.9 (3)
C20—C16—C21—C25	74.3 (5)	C11—C15—Fe2—C18	−34.0 (7)
Fe2—C16—C21—C25	−18.1 (7)	C14—C15—Fe2—C18	−153.9 (6)
C17—C16—C21—C22	73.7 (5)	C11—C15—Fe2—C17	−80.4 (3)
C20—C16—C21—C22	−95.1 (5)	C14—C15—Fe2—C17	159.7 (2)
Fe2—C16—C21—C22	172.6 (3)	C14—C15—Fe2—C11	−119.9 (3)
C17—C16—C21—Fe3	−18.4 (6)	C11—C15—Fe2—C16	−123.1 (2)
C20—C16—C21—Fe3	172.9 (3)	C14—C15—Fe2—C16	117.0 (3)
Fe2—C16—C21—Fe3	80.5 (5)	C16—C17—Fe2—C19	82.7 (2)
C25—C21—C22—C23	0.1 (5)	C18—C17—Fe2—C19	−37.8 (2)
C16—C21—C22—C23	171.5 (4)	C16—C17—Fe2—C13	147.5 (4)
Fe3—C21—C22—C23	−59.4 (3)	C18—C17—Fe2—C13	27.1 (5)
C25—C21—C22—Fe3	59.5 (3)	C16—C17—Fe2—C14	−26.9 (6)
C16—C21—C22—Fe3	−129.2 (4)	C18—C17—Fe2—C14	−147.3 (5)
C21—C22—C23—C24	0.3 (5)	C16—C17—Fe2—C20	38.3 (2)
Fe3—C22—C23—C24	−59.8 (3)	C18—C17—Fe2—C20	−82.1 (2)
C21—C22—C23—Fe3	60.1 (3)	C16—C17—Fe2—C12	−166.8 (2)
C22—C23—C24—C25	−0.6 (5)	C18—C17—Fe2—C12	72.7 (3)
Fe3—C23—C24—C25	−60.6 (3)	C16—C17—Fe2—C18	120.4 (3)
C22—C23—C24—Fe3	60.0 (3)	C16—C17—Fe2—C15	−77.0 (3)
C22—C21—C25—C24	−0.5 (4)	C18—C17—Fe2—C15	162.6 (2)
C16—C21—C25—C24	−171.3 (4)	C16—C17—Fe2—C11	−121.2 (2)
Fe3—C21—C25—C24	58.2 (3)	C18—C17—Fe2—C11	118.4 (2)
C22—C21—C25—Fe3	−58.7 (3)	C18—C17—Fe2—C16	−120.4 (3)
C16—C21—C25—Fe3	130.5 (4)	C15—C11—Fe2—C19	−145.4 (4)
C23—C24—C25—C21	0.6 (5)	C12—C11—Fe2—C19	−26.4 (6)
Fe3—C24—C25—C21	−59.3 (3)	C6—C11—Fe2—C19	91.9 (6)
C23—C24—C25—Fe3	59.9 (3)	C15—C11—Fe2—C13	−81.0 (3)
C5—C1—C26—C27	−109.1 (5)	C12—C11—Fe2—C13	38.0 (3)
C2—C1—C26—C27	79.2 (5)	C6—C11—Fe2—C13	156.4 (4)
Fe1—C1—C26—C27	−11.9 (6)	C15—C11—Fe2—C14	−37.4 (2)
C5—C1—C26—C30	79.4 (5)	C12—C11—Fe2—C14	81.6 (3)
C2—C1—C26—C30	−92.2 (5)	C6—C11—Fe2—C14	−160.0 (4)
Fe1—C1—C26—C30	176.7 (3)	C15—C11—Fe2—C20	32.4 (6)
C5—C1—C26—Fe3	−11.8 (7)	C12—C11—Fe2—C20	151.4 (5)
C2—C1—C26—Fe3	176.5 (3)	C6—C11—Fe2—C20	−90.2 (6)
Fe1—C1—C26—Fe3	85.5 (5)	C15—C11—Fe2—C12	−119.0 (3)
C30—C26—C27—C28	0.9 (4)	C6—C11—Fe2—C12	118.4 (5)
C1—C26—C27—C28	−171.6 (4)	C15—C11—Fe2—C18	169.3 (2)
Fe3—C26—C27—C28	58.7 (3)	C12—C11—Fe2—C18	−71.7 (3)
C30—C26—C27—Fe3	−57.8 (3)	C6—C11—Fe2—C18	46.7 (4)
C1—C26—C27—Fe3	129.6 (4)	C12—C11—Fe2—C15	119.0 (3)
C26—C27—C28—C29	0.2 (5)	C6—C11—Fe2—C15	−122.6 (5)
Fe3—C27—C28—C29	60.1 (3)	C15—C11—Fe2—C17	124.2 (2)
C26—C27—C28—Fe3	−59.9 (3)	C12—C11—Fe2—C17	−116.8 (3)
C27—C28—C29—C30	−1.3 (5)	C6—C11—Fe2—C17	1.6 (4)
Fe3—C28—C29—C30	59.2 (3)	C15—C11—Fe2—C16	79.8 (3)
C27—C28—C29—Fe3	−60.5 (3)	C12—C11—Fe2—C16	−161.2 (2)
C28—C29—C30—C26	1.8 (5)	C6—C11—Fe2—C16	−42.8 (5)
Fe3—C29—C30—C26	61.2 (3)	C17—C16—Fe2—C19	−80.5 (3)
C28—C29—C30—Fe3	−59.4 (3)	C20—C16—Fe2—C19	38.0 (3)
C27—C26—C30—C29	−1.7 (4)	C21—C16—Fe2—C19	155.3 (5)
C1—C26—C30—C29	171.2 (4)	C17—C16—Fe2—C13	−147.6 (4)
Fe3—C26—C30—C29	−60.3 (3)	C20—C16—Fe2—C13	−29.0 (5)
C27—C26—C30—Fe3	58.6 (3)	C21—C16—Fe2—C13	88.3 (6)
C1—C26—C30—Fe3	−128.5 (4)	C17—C16—Fe2—C14	170.7 (2)
C10—C9—Fe1—C3	120.1 (3)	C20—C16—Fe2—C14	−70.7 (3)
C8—C9—Fe1—C3	−121.1 (3)	C21—C16—Fe2—C14	46.6 (5)
C10—C9—Fe1—C8	−118.8 (4)	C17—C16—Fe2—C20	−118.6 (3)
C8—C9—Fe1—C10	118.8 (4)	C21—C16—Fe2—C20	117.3 (5)
C10—C9—Fe1—C4	80.3 (3)	C17—C16—Fe2—C12	38.3 (6)
C8—C9—Fe1—C4	−160.9 (2)	C20—C16—Fe2—C12	156.8 (5)
C10—C9—Fe1—C2	160.4 (3)	C21—C16—Fe2—C12	−85.9 (7)
C8—C9—Fe1—C2	−80.7 (3)	C17—C16—Fe2—C18	−37.0 (2)
C10—C9—Fe1—C7	−80.8 (3)	C20—C16—Fe2—C18	81.6 (3)
C8—C9—Fe1—C7	38.0 (2)	C21—C16—Fe2—C18	−161.1 (5)
C10—C9—Fe1—C5	58.1 (5)	C17—C16—Fe2—C15	126.2 (2)
C8—C9—Fe1—C5	176.9 (4)	C20—C16—Fe2—C15	−115.2 (3)
C10—C9—Fe1—C1	−176.2 (4)	C21—C16—Fe2—C15	2.1 (5)
C8—C9—Fe1—C1	−57.3 (5)	C20—C16—Fe2—C17	118.6 (3)
C10—C9—Fe1—C6	−37.4 (3)	C21—C16—Fe2—C17	−124.1 (5)
C8—C9—Fe1—C6	81.4 (3)	C17—C16—Fe2—C11	82.9 (3)
C4—C3—Fe1—C9	−120.8 (3)	C20—C16—Fe2—C11	−158.5 (3)
C2—C3—Fe1—C9	120.0 (3)	C21—C16—Fe2—C11	−41.3 (5)
C4—C3—Fe1—C8	−160.6 (2)	C22—C23—Fe3—C24	−118.7 (4)
C2—C3—Fe1—C8	80.2 (3)	C22—C23—Fe3—C28	83.2 (3)
C4—C3—Fe1—C10	−81.2 (3)	C24—C23—Fe3—C28	−158.1 (3)
C2—C3—Fe1—C10	159.7 (2)	C22—C23—Fe3—C30	162.3 (3)
C2—C3—Fe1—C4	−119.1 (3)	C24—C23—Fe3—C30	−78.9 (3)
C4—C3—Fe1—C2	119.1 (3)	C22—C23—Fe3—C29	122.9 (3)
C4—C3—Fe1—C7	177.7 (4)	C24—C23—Fe3—C29	−118.4 (3)
C2—C3—Fe1—C7	58.6 (5)	C24—C23—Fe3—C22	118.7 (4)
C4—C3—Fe1—C5	37.6 (2)	C22—C23—Fe3—C27	60.5 (5)
C2—C3—Fe1—C5	−81.6 (3)	C24—C23—Fe3—C27	179.2 (3)
C4—C3—Fe1—C1	80.9 (2)	C22—C23—Fe3—C25	−81.0 (3)
C2—C3—Fe1—C1	−38.2 (2)	C24—C23—Fe3—C25	37.8 (2)
C4—C3—Fe1—C6	−56.9 (5)	C22—C23—Fe3—C21	−37.7 (3)
C2—C3—Fe1—C6	−176.1 (4)	C24—C23—Fe3—C21	81.0 (3)
C7—C8—Fe1—C9	118.6 (4)	C22—C23—Fe3—C26	−178.6 (4)
C9—C8—Fe1—C3	73.7 (3)	C24—C23—Fe3—C26	−59.9 (6)
C7—C8—Fe1—C3	−167.7 (2)	C25—C24—Fe3—C23	119.0 (4)
C9—C8—Fe1—C10	−37.7 (2)	C23—C24—Fe3—C28	44.7 (5)
C7—C8—Fe1—C10	80.9 (2)	C25—C24—Fe3—C28	163.7 (3)
C9—C8—Fe1—C4	42.9 (5)	C23—C24—Fe3—C30	117.9 (3)
C7—C8—Fe1—C4	161.4 (3)	C25—C24—Fe3—C30	−123.1 (3)
C9—C8—Fe1—C2	115.9 (3)	C23—C24—Fe3—C29	75.6 (3)
C7—C8—Fe1—C2	−125.5 (2)	C25—C24—Fe3—C29	−165.4 (2)
C9—C8—Fe1—C7	−118.6 (4)	C23—C24—Fe3—C22	−37.8 (2)
C9—C8—Fe1—C5	−176.1 (5)	C25—C24—Fe3—C22	81.2 (3)
C7—C8—Fe1—C5	−57.5 (6)	C23—C24—Fe3—C27	−178.8 (5)
C9—C8—Fe1—C1	156.9 (2)	C25—C24—Fe3—C27	−59.8 (7)
C7—C8—Fe1—C1	−84.5 (3)	C23—C24—Fe3—C25	−119.0 (4)
C9—C8—Fe1—C6	−81.8 (3)	C23—C24—Fe3—C21	−82.1 (3)
C7—C8—Fe1—C6	36.8 (2)	C25—C24—Fe3—C21	36.9 (2)
C6—C10—Fe1—C9	−120.1 (4)	C23—C24—Fe3—C26	157.6 (2)
C9—C10—Fe1—C3	−74.4 (3)	C25—C24—Fe3—C26	−83.4 (3)
C6—C10—Fe1—C3	165.5 (3)	C29—C28—Fe3—C23	75.8 (3)
C9—C10—Fe1—C8	38.0 (3)	C27—C28—Fe3—C23	−165.3 (2)
C6—C10—Fe1—C8	−82.0 (3)	C29—C28—Fe3—C24	45.0 (5)
C9—C10—Fe1—C4	−116.4 (3)	C27—C28—Fe3—C24	163.8 (3)
C6—C10—Fe1—C4	123.5 (3)	C29—C28—Fe3—C30	−37.8 (2)
C9—C10—Fe1—C2	−42.7 (5)	C27—C28—Fe3—C30	81.1 (3)
C6—C10—Fe1—C2	−162.8 (4)	C27—C28—Fe3—C29	118.9 (4)
C9—C10—Fe1—C7	82.1 (3)	C29—C28—Fe3—C22	117.8 (3)
C6—C10—Fe1—C7	−38.0 (2)	C27—C28—Fe3—C22	−123.3 (3)
C9—C10—Fe1—C5	−156.4 (3)	C29—C28—Fe3—C27	−118.9 (4)
C6—C10—Fe1—C5	83.5 (3)	C29—C28—Fe3—C25	−178.7 (5)
C9—C10—Fe1—C1	175.2 (5)	C27—C28—Fe3—C25	−59.9 (7)
C6—C10—Fe1—C1	55.1 (6)	C29—C28—Fe3—C21	157.9 (2)
C9—C10—Fe1—C6	120.1 (4)	C27—C28—Fe3—C21	−83.3 (3)
C3—C4—Fe1—C9	73.9 (3)	C29—C28—Fe3—C26	−82.0 (3)
C5—C4—Fe1—C9	−166.9 (2)	C27—C28—Fe3—C26	36.9 (2)
C5—C4—Fe1—C3	119.2 (3)	C29—C30—Fe3—C23	−72.2 (3)
C3—C4—Fe1—C8	43.4 (5)	C26—C30—Fe3—C23	169.8 (3)
C5—C4—Fe1—C8	162.6 (4)	C29—C30—Fe3—C24	−113.6 (3)
C3—C4—Fe1—C10	115.5 (2)	C26—C30—Fe3—C24	128.4 (3)
C5—C4—Fe1—C10	−125.3 (2)	C29—C30—Fe3—C28	37.2 (3)
C3—C4—Fe1—C2	−38.0 (2)	C26—C30—Fe3—C28	−80.7 (3)
C5—C4—Fe1—C2	81.2 (2)	C26—C30—Fe3—C29	−118.0 (4)
C3—C4—Fe1—C7	−177.3 (4)	C29—C30—Fe3—C22	−43.1 (6)
C5—C4—Fe1—C7	−58.2 (6)	C26—C30—Fe3—C22	−161.0 (4)
C3—C4—Fe1—C5	−119.2 (3)	C29—C30—Fe3—C27	81.0 (3)
C3—C4—Fe1—C1	−82.3 (2)	C26—C30—Fe3—C27	−36.9 (2)
C5—C4—Fe1—C1	36.8 (2)	C29—C30—Fe3—C25	−154.2 (3)
C3—C4—Fe1—C6	156.4 (2)	C26—C30—Fe3—C25	87.9 (3)
C5—C4—Fe1—C6	−84.5 (3)	C29—C30—Fe3—C21	177.4 (4)
C3—C2—Fe1—C9	−74.8 (3)	C26—C30—Fe3—C21	59.5 (6)
C1—C2—Fe1—C9	166.6 (3)	C29—C30—Fe3—C26	118.0 (4)
C1—C2—Fe1—C3	−118.7 (4)	C28—C29—Fe3—C23	−118.0 (3)
C3—C2—Fe1—C8	−116.7 (3)	C30—C29—Fe3—C23	122.6 (3)
C1—C2—Fe1—C8	124.6 (3)	C28—C29—Fe3—C24	−158.0 (3)
C3—C2—Fe1—C10	−44.7 (5)	C30—C29—Fe3—C24	82.6 (3)
C1—C2—Fe1—C10	−163.4 (4)	C30—C29—Fe3—C28	−119.4 (4)
C3—C2—Fe1—C4	37.6 (2)	C28—C29—Fe3—C30	119.4 (4)
C1—C2—Fe1—C4	−81.0 (3)	C28—C29—Fe3—C22	−79.1 (3)
C3—C2—Fe1—C7	−157.0 (2)	C30—C29—Fe3—C22	161.5 (3)
C1—C2—Fe1—C7	84.4 (3)	C28—C29—Fe3—C27	37.9 (2)
C3—C2—Fe1—C5	81.2 (3)	C30—C29—Fe3—C27	−81.6 (3)
C1—C2—Fe1—C5	−37.5 (2)	C28—C29—Fe3—C25	179.2 (3)
C3—C2—Fe1—C1	118.7 (4)	C30—C29—Fe3—C25	59.8 (5)
C3—C2—Fe1—C6	174.8 (5)	C28—C29—Fe3—C21	−58.1 (6)
C1—C2—Fe1—C6	56.2 (6)	C30—C29—Fe3—C21	−177.5 (4)
C6—C7—Fe1—C9	82.2 (3)	C28—C29—Fe3—C26	81.0 (3)
C8—C7—Fe1—C9	−38.1 (2)	C30—C29—Fe3—C26	−38.5 (2)
C6—C7—Fe1—C3	149.8 (4)	C21—C22—Fe3—C23	−119.6 (4)
C8—C7—Fe1—C3	29.6 (5)	C23—C22—Fe3—C24	37.9 (3)
C6—C7—Fe1—C8	120.3 (3)	C21—C22—Fe3—C24	−81.7 (3)
C6—C7—Fe1—C10	38.5 (2)	C23—C22—Fe3—C28	−112.9 (3)
C8—C7—Fe1—C10	−81.8 (3)	C21—C22—Fe3—C28	127.5 (3)
C6—C7—Fe1—C4	−34.8 (6)	C23—C22—Fe3—C30	−40.7 (6)
C8—C7—Fe1—C4	−155.1 (4)	C21—C22—Fe3—C30	−160.3 (4)
C6—C7—Fe1—C2	−167.2 (2)	C23—C22—Fe3—C29	−71.7 (4)
C8—C7—Fe1—C2	72.6 (3)	C21—C22—Fe3—C29	168.7 (3)
C6—C7—Fe1—C5	−79.0 (3)	C23—C22—Fe3—C27	−153.5 (3)
C8—C7—Fe1—C5	160.7 (3)	C21—C22—Fe3—C27	86.9 (3)
C6—C7—Fe1—C1	−122.2 (2)	C23—C22—Fe3—C25	81.8 (3)
C8—C7—Fe1—C1	117.5 (3)	C21—C22—Fe3—C25	−37.8 (2)
C8—C7—Fe1—C6	−120.3 (3)	C23—C22—Fe3—C21	119.6 (4)
C4—C5—Fe1—C9	30.7 (5)	C23—C22—Fe3—C26	178.6 (4)
C1—C5—Fe1—C9	151.1 (4)	C21—C22—Fe3—C26	58.9 (6)
C4—C5—Fe1—C3	−37.8 (2)	C26—C27—Fe3—C23	151.7 (4)
C1—C5—Fe1—C3	82.6 (2)	C28—C27—Fe3—C23	31.9 (5)
C4—C5—Fe1—C8	−155.9 (5)	C26—C27—Fe3—C24	−30.2 (7)
C1—C5—Fe1—C8	−35.5 (6)	C28—C27—Fe3—C24	−149.9 (6)
C4—C5—Fe1—C10	72.7 (3)	C26—C27—Fe3—C28	119.7 (4)
C1—C5—Fe1—C10	−167.0 (2)	C26—C27—Fe3—C30	37.9 (2)
C1—C5—Fe1—C4	120.4 (3)	C28—C27—Fe3—C30	−81.8 (3)
C4—C5—Fe1—C2	−82.1 (2)	C26—C27—Fe3—C29	82.1 (3)
C1—C5—Fe1—C2	38.3 (2)	C28—C27—Fe3—C29	−37.6 (3)
C4—C5—Fe1—C7	160.0 (2)	C26—C27—Fe3—C22	−165.8 (2)
C1—C5—Fe1—C7	−79.6 (3)	C28—C27—Fe3—C22	74.5 (3)
C4—C5—Fe1—C1	−120.4 (3)	C26—C27—Fe3—C25	−77.7 (3)
C4—C5—Fe1—C6	117.0 (2)	C28—C27—Fe3—C25	162.6 (3)
C1—C5—Fe1—C6	−122.7 (2)	C26—C27—Fe3—C21	−120.6 (2)
C5—C1—Fe1—C9	−150.9 (4)	C28—C27—Fe3—C21	119.7 (3)
C2—C1—Fe1—C9	−32.0 (6)	C28—C27—Fe3—C26	−119.7 (4)
C26—C1—Fe1—C9	84.7 (6)	C21—C25—Fe3—C23	82.3 (3)
C5—C1—Fe1—C3	−80.8 (3)	C24—C25—Fe3—C23	−37.7 (3)
C2—C1—Fe1—C3	38.1 (2)	C21—C25—Fe3—C24	120.0 (4)
C26—C1—Fe1—C3	154.8 (4)	C21—C25—Fe3—C28	−29.8 (7)
C5—C1—Fe1—C8	167.5 (2)	C24—C25—Fe3—C28	−149.8 (6)
C2—C1—Fe1—C8	−73.7 (3)	C21—C25—Fe3—C30	−165.5 (2)
C26—C1—Fe1—C8	43.0 (4)	C24—C25—Fe3—C30	74.5 (3)
C5—C1—Fe1—C10	37.3 (6)	C21—C25—Fe3—C29	152.1 (3)
C2—C1—Fe1—C10	156.2 (5)	C24—C25—Fe3—C29	32.1 (5)
C26—C1—Fe1—C10	−87.1 (7)	C21—C25—Fe3—C22	38.5 (2)
C5—C1—Fe1—C4	−37.0 (2)	C24—C25—Fe3—C22	−81.5 (3)
C2—C1—Fe1—C4	81.9 (3)	C21—C25—Fe3—C27	−77.3 (3)
C26—C1—Fe1—C4	−161.4 (4)	C24—C25—Fe3—C27	162.7 (3)
C5—C1—Fe1—C2	−118.9 (3)	C24—C25—Fe3—C21	−120.0 (4)
C26—C1—Fe1—C2	116.7 (5)	C21—C25—Fe3—C26	−120.2 (2)
C5—C1—Fe1—C7	123.0 (2)	C24—C25—Fe3—C26	119.8 (3)
C2—C1—Fe1—C7	−118.1 (2)	C25—C21—Fe3—C23	−81.0 (3)
C26—C1—Fe1—C7	−1.4 (4)	C22—C21—Fe3—C23	37.2 (3)
C2—C1—Fe1—C5	118.9 (3)	C16—C21—Fe3—C23	154.5 (5)
C26—C1—Fe1—C5	−124.4 (5)	C25—C21—Fe3—C24	−37.3 (2)
C5—C1—Fe1—C6	79.6 (3)	C22—C21—Fe3—C24	80.9 (3)
C2—C1—Fe1—C6	−161.5 (2)	C16—C21—Fe3—C24	−161.8 (5)
C26—C1—Fe1—C6	−44.8 (5)	C25—C21—Fe3—C28	170.7 (2)
C7—C6—Fe1—C9	−81.1 (3)	C22—C21—Fe3—C28	−71.1 (3)
C10—C6—Fe1—C9	37.0 (3)	C16—C21—Fe3—C28	46.2 (5)
C11—C6—Fe1—C9	154.8 (4)	C25—C21—Fe3—C30	38.2 (6)
C7—C6—Fe1—C3	−151.6 (4)	C22—C21—Fe3—C30	156.4 (4)
C10—C6—Fe1—C3	−33.5 (5)	C16—C21—Fe3—C30	−86.3 (6)
C11—C6—Fe1—C3	84.3 (5)	C25—C21—Fe3—C29	−146.5 (5)
C7—C6—Fe1—C8	−37.2 (2)	C22—C21—Fe3—C29	−28.3 (6)
C10—C6—Fe1—C8	80.9 (3)	C16—C21—Fe3—C29	88.9 (6)
C11—C6—Fe1—C8	−161.3 (4)	C25—C21—Fe3—C22	−118.2 (4)
C7—C6—Fe1—C10	−118.1 (3)	C16—C21—Fe3—C22	117.3 (5)
C11—C6—Fe1—C10	117.8 (5)	C25—C21—Fe3—C27	126.3 (2)
C7—C6—Fe1—C4	167.4 (2)	C22—C21—Fe3—C27	−115.5 (3)
C10—C6—Fe1—C4	−74.5 (3)	C16—C21—Fe3—C27	1.8 (4)
C11—C6—Fe1—C4	43.3 (4)	C22—C21—Fe3—C25	118.2 (4)
C7—C6—Fe1—C2	36.9 (6)	C16—C21—Fe3—C25	−124.5 (5)
C10—C6—Fe1—C2	155.0 (5)	C25—C21—Fe3—C26	82.9 (3)
C11—C6—Fe1—C2	−87.2 (7)	C22—C21—Fe3—C26	−158.9 (3)
C10—C6—Fe1—C7	118.1 (3)	C16—C21—Fe3—C26	−41.6 (5)
C11—C6—Fe1—C7	−124.1 (4)	C27—C26—Fe3—C23	−145.1 (5)
C7—C6—Fe1—C5	123.4 (2)	C30—C26—Fe3—C23	−25.6 (6)
C10—C6—Fe1—C5	−118.5 (3)	C1—C26—Fe3—C23	91.4 (6)
C11—C6—Fe1—C5	−0.7 (4)	C27—C26—Fe3—C24	170.5 (2)
C7—C6—Fe1—C1	80.3 (3)	C30—C26—Fe3—C24	−70.0 (3)
C10—C6—Fe1—C1	−161.6 (3)	C1—C26—Fe3—C24	47.0 (4)
C11—C6—Fe1—C1	−43.8 (5)	C27—C26—Fe3—C28	−37.5 (2)
C18—C19—Fe2—C13	−120.8 (3)	C30—C26—Fe3—C28	82.0 (3)
C20—C19—Fe2—C13	120.2 (3)	C1—C26—Fe3—C28	−161.1 (4)
C18—C19—Fe2—C14	−160.3 (3)	C27—C26—Fe3—C30	−119.5 (3)
C20—C19—Fe2—C14	80.7 (3)	C1—C26—Fe3—C30	117.0 (5)
C18—C19—Fe2—C20	119.0 (4)	C27—C26—Fe3—C29	−81.0 (3)
C18—C19—Fe2—C12	−81.5 (3)	C30—C26—Fe3—C29	38.4 (3)
C20—C19—Fe2—C12	159.5 (2)	C1—C26—Fe3—C29	155.4 (5)
C20—C19—Fe2—C18	−119.0 (4)	C27—C26—Fe3—C22	37.6 (6)
C18—C19—Fe2—C15	178.5 (3)	C30—C26—Fe3—C22	157.0 (5)
C20—C19—Fe2—C15	59.5 (5)	C1—C26—Fe3—C22	−86.0 (6)
C18—C19—Fe2—C17	37.8 (2)	C30—C26—Fe3—C27	119.5 (3)
C20—C19—Fe2—C17	−81.2 (3)	C1—C26—Fe3—C27	−123.6 (5)
C18—C19—Fe2—C11	−62.1 (5)	C27—C26—Fe3—C25	126.0 (2)
C20—C19—Fe2—C11	178.9 (4)	C30—C26—Fe3—C25	−114.6 (3)
C18—C19—Fe2—C16	81.1 (3)	C1—C26—Fe3—C25	2.4 (4)
C20—C19—Fe2—C16	−37.9 (2)	C27—C26—Fe3—C21	82.0 (3)
C14—C13—Fe2—C19	−118.9 (3)	C30—C26—Fe3—C21	−158.6 (3)
C12—C13—Fe2—C19	121.8 (3)	C1—C26—Fe3—C21	−41.6 (5)
C12—C13—Fe2—C14	−119.3 (4)		

### Hydrogen-bond geometry (Å, º)

Cg1 and Cg4 are the centroids of the C1–C5 and C16–C20 rings, respectively.

**Table:**

*D*—H···*A*	*D*—H	H···*A*	*D*···*A*	*D*—H···*A*
C30—H30···*Cg*1^i^	0.98	2.89	3.668 (5)	137
C28—H28···*Cg*4^ii^	0.98	2.70	3.601 (6)	153

Symmetry codes: (i) −*x*+1, −*y*+1, −*z*+1; (ii) −*x*+1, −*y*, −*z*.
