# Supplementary figures and images for: Crystal structure of cyclic tris­(ferrocene-1,1′-di­yl)
Source: Acta Crystallogr Sect E Struct Rep Online. 2014 Aug 1;70(Pt 9):m318–9. doi: 10.1107/S1600536814017346 (PMC4186187; doi:10.1107/S1600536814017346)

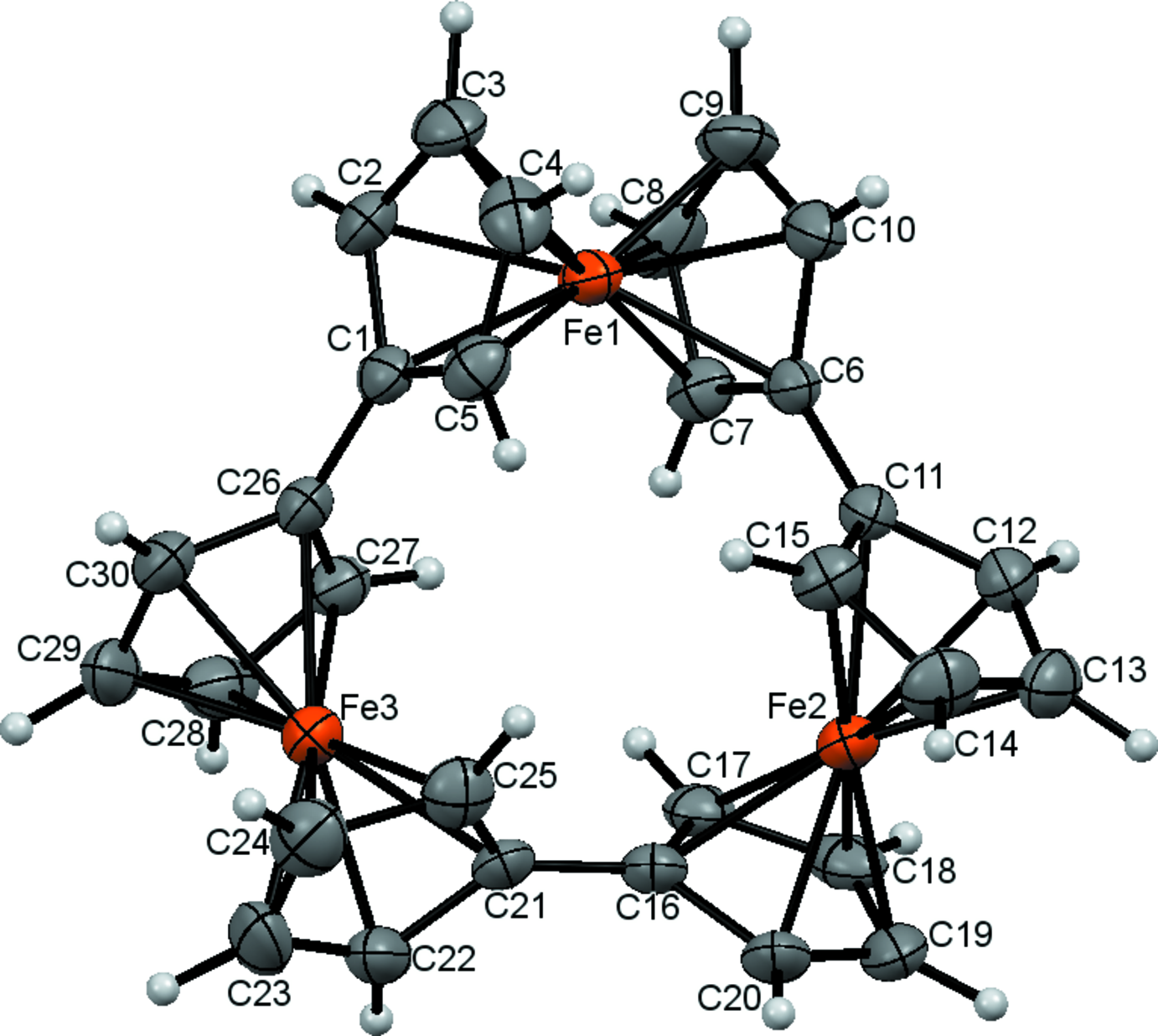

Supplement: Supplementary file 4 [file e-70-0m318-fig1.tif]
